# Novel strategies for nanovaccine application in breast cancer treatment

**DOI:** 10.3389/fphar.2025.1691991

**Published:** 2025-10-16

**Authors:** Huan Liu, Ning Ma

**Affiliations:** Department of Breast Surgery, Baoding NO.1 Central Hospital, Baoding, Hebei, China

**Keywords:** breast cancer, nanovaccines, neoantigens, microenvironment-responsive, immunotherapy combined therapy

## Abstract

Breast cancer (BC), as the most common malignant tumor among women globally, faces numerous challenges in treatment. Currently, although the combination of surgical resection with various treatment modalities such as chemotherapy, radiotherapy, and immunotherapy can improve patient survival rates, most BC patients are diagnosed at an advanced stage. Additionally, traditional small-molecule targeted anticancer drugs used during chemotherapy suffer from issues such as low target selectivity and frequent occurrence of acquired resistance, leading to suboptimal therapeutic outcomes. In recent years, tumor immunotherapy has achieved significant progress. Notably, cancer vaccines, as a strategy within tumor immunotherapy, represent a promising therapeutic approach by inducing tumor-specific immune responses. However, cancer vaccines also face several limitations, such as the strong systemic toxicity of immune adjuvants and the low immunogenicity of tumor antigens. An ideal cancer vaccine should sufficiently stimulate the body to generate a potent tumor-specific immune response while exhibiting low side effects. With innovations in nanotechnology within the medical field, nanotechnology can help overcome the limitations of traditional vaccines in the realm of vaccination. This review summarizes various nanomaterial carriers applied in the manufacturing of nanovaccines. These nanomaterials exhibit excellent biocompatibility, adjuvant activity, and immunogenicity. Furthermore, we discuss and summarize preparation strategies to enhance antitumor immune efficacy, including the use of tumor neoantigens to improve antigen presentation, the application of tumor cell membrane-based biomimetic nanotechnology to promote targeted delivery of nanovaccines, nanovaccine delivery platforms targeting the tumor microenvironment (TME), and novel strategies for combined application with nanovaccines. As an emerging strategy for BC immunotherapy, nanovaccines offer a novel approach to address the challenges of BC treatment and post-surgical recurrence by enabling precise delivery of tumor antigens, efficient activation of immune responses, and remodeling of the tumor microenvironment.

## 1 Introduction

Breast cancer (BC), the most common malignant tumor among women worldwide, presents numerous treatment challenges, particularly in the case of triple-negative breast cancer (TNBC), its most aggressive subtype. TNBC accounts for approximately 15%–20% of all BC cases and is characterized by the absence of estrogen receptor (ER), progesterone receptor (PR), and human epidermal growth factor receptor 2 (HER2) expression (Frangou et al., 2014). This renders endocrine therapy and HER2-targeted treatments ineffective (Derakhshan and Reis-Filho, 2022; Li et al., 2022). TNBC is associated with a high risk of recurrence, a propensity for early metastasis, and a poor prognosis, resulting in a significantly lower 5-year survival rate compared to other breast cancer subtypes. Globally, one woman is diagnosed with BC every 14 s. In 2022 alone, approximately 2.3 million women were diagnosed with the disease, and 670,000 died from it, with TNBC being an important cause of this mortality (Katsura et al., 2022; Xiong et al., 2025). Currently, although the combination of surgical resection with various treatment modalities such as chemotherapy, radiotherapy, and immunotherapy can improve patient survival rates, most BC patients are diagnosed at an advanced stage. Additionally, traditional small-molecule targeted anticancer drugs used during chemotherapy suffer from issues such as low target selectivity and frequent occurrence of acquired resistance, leading to suboptimal therapeutic outcomes.

In recent years, significant progress has been made in cancer immunotherapy. However, BC, particularly TNBC, exhibits relatively low immunogenicity and possesses characteristics of a “cold tumor” microenvironment. This manifests as insufficient T-cell infiltration, increased infiltration of immunosuppressive cells (such as M2-type macrophages and regulatory T cells), and high expression of various immunosuppressive molecules. Transforming these “cold tumors” into “hot tumors” by enhancing immune cell infiltration and activity is key to improving immunotherapy efficacy. Nanovaccine technology offers a novel approach to addressing these challenges by precisely delivering tumor antigens and immune adjuvants to activate specific immune responses (Das and Ali, 2021; Qin et al., 2021). In the field of vaccination, nanotechnology also offers solutions to improve conventional vaccines. Nanotechnology refers to the science related to nanoparticles (NPs), defined as particles sized between 10 and 100 nm, extending up to 1,000 nm in certain cases. Nanomaterials have become common carriers in nanovaccine preparation due to their small size, structural stability, large surface area and cavity volume, modifiable surfaces, and ability to protect and deliver multiple antigens efficiently to target cells. Additionally, they offer advantages in humans including safety with low toxicity, high delivery efficiency, and strong immunogenicity.

In this review, we systematically summarize various nanomaterial carriers used in the development of nanovaccines, such as liposome NPs, inorganic NPs, polymer NPs, and biomimetic membrane NPs. We also summarize preparation strategies to enhance antitumor immune efficacy. These include the application of tumor neoantigens to improve antigen presentation, the use of tumor cell membrane biomimetic nanotechnology to promote targeted delivery of nanovaccines, nanovaccine delivery platforms targeting the tumor microenvironment (TME), and novel strategies for combination therapies with nanovaccines. These innovative approaches to nanovaccine preparation are expected to have a profound impact on the treatment of cancers such as BC.

## 2 Advantages of nanovaccines in cancer immunotherapy

Tumor immunotherapy is a promising therapeutic approach that inhibits tumor progression by stimulating the body to mount an anti-tumor immune response. Among the various tumor immunotherapy strategies, immune checkpoint inhibitors, such as anti-PD-1 antibodies, have been widely used in the clinical treatment of tumors. Cancer vaccines (CVs), as another strategy for tumor immunotherapy, can induce tumor-specific immune responses and represent a highly promising treatment modality. CVs typically consist of tumor antigens, adjuvants, and a delivery system. They work by introducing specific tumor antigens to stimulate the body to generate robust humoral and cellular immune responses that recognize and kill tumor cells. This approach can effectively reduce non-specific killing and adverse reactions, while establishing long-lasting protective immune memory. CVs have shown great potential in treating patients with low response rates to other standard therapies or immunotherapies. Following administration, tumor antigens are recognized and captured by specialized antigen-presenting cells (APCs) within the body. These APCs then migrate to the draining lymph nodes, where they induce the proliferation and differentiation of T lymphocytes and B lymphocytes. Ultimately, cancer cells expressing the target antigens are killed and eliminated either by cytokines secreted by cytotoxic T lymphocytes (CTLs) or by antibodies secreted by plasma cells.

However, cancer vaccines also face several limitations, such as the strong systemic toxicity of immune adjuvants and the low immunogenicity of tumor antigens. An ideal cancer vaccine should sufficiently stimulate the body to elicit potent tumor-specific immune responses while minimizing adverse effects. During immune activation, adequate antigenic material must be captured by APCs like dendritic cells (DCs), and stimulatory adjuvants should concurrently activate these DCs. Once activated by adjuvants, DCs present antigens to T lymphocytes, activating bot CTLs and helper T lymphocytes. Concurrently, B cells are indirectly activated to produce antibodies that drive humoral immunity. DCs can also activate natural killer (NK) cells through IFN-α secretion. Ultimately, CTLs and NK cells migrate to tumor sites, overcoming the immunosuppressive tumor microenvironment to eliminate cancer cells. Throughout this antitumor immune cascade, the vaccine’s systemic toxicity must remain controllable. As APCs, DCs play a pivotal role in inducing antitumor immunity. Without sufficient maturation signals, antigen presentation by steady-state DCs may induce T cell tolerance, thereby hindering antitumor responses. To develop safe and effective cancer vaccines, all these factors must be integrated into the vaccine design process. In short, the antitumor immunostimulatory efficiency of the two fundamental components of cancer vaccines—antigenic material and adjuvants—should be enhanced. Antigenic material can be classified into tumor-associated antigens (TAAs) and tumor-specific antigens (TSAs), the latter also known as neoantigens. Among these, TSAs exhibit tumor specificity, ensuring the induction of tumor-specific immune responses while reducing the systemic immunotoxicity of cancer vaccines. Immunostimulatory adjuvants and drug delivery systems, serving as vaccine adjuvants, are pivotal for improving the immunostimulatory efficiency of cancer vaccines. Through active immunization, cancer vaccines activate tumor antigen-specific T cell responses, enhance immune recognition and clearance of cancer cells, establish immunological memory to reduce cancer recurrence risk, extend patient survival, and minimize non-specific cytotoxicity. They represent a highly promising frontier in next-generation immunotherapy. Therefore, an ideal cancer vaccine should deliver sufficient antigen sources to activate anti-tumor immune responses, thereby inducing both innate and adaptive immune responses while simultaneously overcoming the immunosuppressive tumor microenvironment.

Today, nanotechnology has been introduced into many areas of medicine and can address numerous healthcare challenges. By employing nanomaterials as vaccine carriers, antigens can be protected from degradation by phagocytes. These carriers also enable binding to specific receptors on target cell surfaces, thereby reducing off-target antigen distribution and enhancing delivery efficiency. Nanovaccines demonstrate the potential for cross-presenting antigens via MHC class I molecules and can activate both humoral and cellular immune systems. Consequently, they prove more effective than traditional vaccines (Warrier et al., 2019; Yao et al., 2023).

## 3 Design strategies for nanovaccines to enhance therapeutic efficiency in breast cancer

The core mechanism of nanovaccines lies in enhancing the presentation efficiency of tumor antigens and initiating specific immune responses. This process involves several key steps: Due to their small size (typically 100–200 nm), nanovaccines are efficiently drained through lymphatic vessels to lymph nodes, where they accumulate and facilitate antigen exposure to immune cells. Subsequently, after being taken up by DCs, nanovaccines promote DC maturation via multiple mechanisms, thereby enhancing the induction of anti-tumor immune responses (Zhang et al., 2019; Das and Ali, 2021).

### 3.1 High-performance nanocarriers

The core of nanovaccines lies in utilizing nanoscale carriers to deliver tumor antigens and immunomodulators. Compared to traditional therapies, antitumor drugs based on nano-delivery systems offer higher efficacy and fewer side effects. Simultaneously, these nanocarrier systems exhibit excellent water solubility, enabling them to serve as carriers for hydrophobic drugs. This enhances the bioavailability of antitumor drugs while reducing the need for organic solvents. Moreover, nanomaterials can overcome the poor chemical stability of certain drugs. Antigens encapsulated within nanocarriers can be more effectively delivered to lymph nodes, thereby improving the capture efficiency by APCs. The inherent immunomodulatory properties of many nanomaterials allow them to function as adjuvants, stimulating immune responses (Bezbaruah et al., 2022). Currently, numerous studies have applied nano-delivery systems to tumor vaccines, demonstrating promising results in both *in vitro* and *in vivo* experiments. Based on material characteristics, nanocarriers commonly used in tumor nanovaccines can be mainly categorized into the following types (Table 1).

**TABLE 1:** Characteristics of nanocarriers for nanovaccine.

Nanocarrier type	Nanomaterials	Advantages	Limitations	Application
Lipid NPs	Solid lipid NPs (SLNs), nanostructured lipid carriers (NLCs)	High biocompatibility, easy surface modification, and capable of carrying multiple antigens	Challenges of large-scale production	mRNA vaccine delivery
Polymeric NPs	Polylactic acid - glycolic acid copolymer (PLGA), chitosan	Controllable release, good degradability and easy functionalization	The drug loading efficiency is unstable	Antigen sustained-release and adjuvant co-delivery
Inorganic NPs	Gold NPs, silica NPs, calcium carbonate NPs	Good stability and surface plasma effect	Poor biodegradability	Photothermal combined therapy
Biomimetic NPs	Bacterial outer membrane vesicles (OMV), tumor cell membranes, dendritic cell membranes	Natural targeting and low immunogenicity	The preparation process is complex	Personalized nanovaccine
	Virus-like particles (VLPs)	The ability to self-assemble, natural targeting	Lacking the genetic material so did not capable of infecting the host cell	Personalized nanovaccine

#### 3.1.1 Lipid NPs

Lipid NPs (LNPs) represent the most extensively researched nanocarriers in nanovaccines. These spherical vesicles are composed of amphiphilic phospholipid bilayers surrounding a hydrophilic core. Compared to other nanoparticles, LNPs exhibit superior biodegradability and biocompatibility, along with enhanced tolerance, penetrability, and fluidity. They also significantly improve the solubility of hydrophobic antigens. Critically, the immune response induced by liposomal nanoparticles is modulated by factors including size, surface charge, and immunogenicity. Nanoparticle size dictates immune activation patterns: LNPs ≈ 100nm preferentially trigger Th2-type immune responses; LNPs > 400nm predominantly activate Th1-type immune responses. Cationic liposomes effectively encapsulate negatively charged antigens. Their positive surface charge facilitates electrostatic interactions with negatively charged surfaces of DCs, enhancing antigen presentation and immune activation. Additionally, liposomes function as intrinsic adjuvants in vaccine formulations, imparting potent immunogenicity. Their surfaces demonstrate high engineerability, enabling customizable immune modulation (Koo et al., 2021; Sunoqrot et al., 2024).

#### 3.1.2 Polymeric NPs

Polymeric NPs offer significant advantages including high biocompatibility, strong biodegradability, ease of fabrication, low systemic toxicity, engineerable design flexibility, and surface modifiability. These particles enhance delivery efficiency by incorporating antigens and adjuvants via multiple strategies—solubilization, encapsulation, or surface adsorption—thereby reducing off-target distribution. However, *in vivo* administration entails risks of particle aggregation and potential toxicity when highly concentrated polymer carriers accumulate under physiological conditions (Mao et al., 2023; Sunoqrot et al., 2024).

Organic polymers offer straightforward fabrication methods, enabling the engineerable production of carriers with tailored dimensions, precise surface charge, and customizable geometries, while allowing fine-tuned surface properties. Commonly used synthetic organic polymers include PLGA, branched polymers, and cationic polymers. Among these, PLGA nanoparticles are the predominant organic nanocarriers for vaccine production. Composed of a copolymer of lactic acid and glycolic acid (both α-hydroxy acids), PLGA undergoes biodegradation *in vivo* by entering the tricarboxylic acid (TCA) cycle for metabolic clearance.

#### 3.1.3 Inorganic NPs

Inorganic nanomaterials offer advantages including superior structural integrity, exceptional batch-to-batch reproducibility, and facile antigen conjugation. Despite their inherently lower biocompatibility and biodegradability, surface functionalization techniques—both physical and chemical—can enhance these properties. Critically, these materials exhibit propensity for bioaccumulation, necessitating rigorous assessment of long-term biosafety concerns associated with *in vivo* retention (Zhou et al., 2023).

#### 3.1.4 Biomimetic nanocarriers

Biomimetic nanocarriers, derived from biological sources—particularly tumor cells—exhibit inherently low toxicity, high degradability, and superior biocompatibility. Both exosomes and outer membrane vesicles (OMVs) represent key categories of biogenic nanocarriers (Li et al., 2021; Zhao et al., 2022). Moreover, cell membrane-engineered biomimetic vaccines confer source cell-derived functionalities onto tumor vaccines, enhancing immune evasion, tumor-homing precision, and antitumor immunity activation. Primary membrane sources for constructing such nanovaccines include red blood cell (RBC) membranes, white blood cell (WBC) membranes, platelet (PLT) membranes, tumor cell membranes and bacterial membranes. These bioinspired platforms represent paradigm-shifting delivery systems in modern cancer nanovaccine design (Zhu et al., 2023; Samanta et al., 2025). Additionally, there are virus-like particles (VLPs). VLPs are hollow protein structures with a regular spatial configuration formed by the repeated stacking and arrangement of viral proteins in space. They can be presented to CD4^+^ T cells by antigen-presenting cells (APCs) via MHC class II molecules, or they can escape lysosomes and be presented to CD8^+^ T cells via MHC class I molecules. This process stimulates and activates cytotoxic T lymphocytes (CTLs) and helper T cells, promoting the secretion of cytokines to kill tumor cells (Nooraei et al., 2021). VLPs exhibit strong immunogenicity but lack viral nucleic acid structures, making them unable to replicate or infect, thus relatively safe. Furthermore, VLPs possess natural targeting properties *in vivo*, allowing them to accumulate at sites where oncogenic viruses gather.

### 3.2 Utilizing tumor neoantigens to enhance antigen presentation functions

The immunogenicity of tumor antigens, combined with the efficient uptake and presentation of vaccines by APCs *in vivo*, are key determinants of vaccine efficacy. In recent years, various bioengineering strategies have been employed to enhance the immunogenicity of tumor cells. For instance, immunogenic cell death (ICD) promotes the release of tumor-associated antigens and induces the ectopic expression of damage-associated molecular patterns (DAMPs), thereby triggering potent antigen-specific immune responses. Leveraging immune cells to recognize neoantigens on the tumor surface and activate the immune system to eliminate tumor cells has emerged as a crucial direction in tumor immunotherapy. These neoantigens serve as promising immunotherapeutic targets due to their high specificity, pronounced foreignness, and potent immunogenicity.

Neoantigens are TSAs formed during tumor development, originating from the expression of mutated genes in tumor cells. Having bypassed central immune tolerance, they exhibit high immunogenicity and can activate CD4^+^ T and CD8^+^ T cells to target tumor cells with precision. Compared to traditional vaccines, neoantigen vaccines induce the production of neoantigen-specific T lymphocytes, demonstrate favorable immune tolerance, and provide long-term immune protection. Building on this property, neoantigen vaccines have become a focal point in recent tumor immunotherapy research. Their core advantage lies in high individualization, enabling both avoidance of damage to normal cells and significant enhancement of anti-tumor efficacy. Consequently, neoantigens serve as ideal targets for therapeutic tumor vaccines and T cell-based adoptive cell therapy (ACT) (Ni et al., 2020; Li et al., 2025). Neoantigen tumor vaccines encompass multiple forms, with current neoantigen nanovaccines for breast cancer primarily including peptide/protein vaccines, nucleic acid vaccines, and neoantigen viral vector vaccines (Table 2).

**TABLE 2:** Utilizing tumor neoantigens to enhance antigen presentation functions.

	Nanovaccines	Vaccine antigen	Adjuvant	Tumor model	Outcome	References
Protein/Peptide-based neoantigen	BAPs	tumor cell-derived autophagosomes (TRAPs)	CpG ODN + ADU-S100	4T1 subcutaneous tumor model of mice	Significant suppression tumor growth; prolonged survival period	Qu et al. (2024)
	Man-PDPM@OVA	OVA (SIINFEKL)	——	4T1 subcutaneous tumor model of mice	Regressed tumor growth and elongated the survival of the tumor-bearing mice	Zhou et al. (2020)
	PhMV	HER2-derived CH401 epitope	CpG ODN	DDHER2 murine model of HER2+ breast cancer	Significant delay in tumor growth; significant prolonged survival period	Hu and Steinmetz (2021)
	nAg-MRDE/Mn+ αPD-1	OVA + Rh2	Mn^2+^	4T1 cell xenograft tumor model of mice	Significant delay in tumor growth; significant prolonged survival period; remodels tumor microenvironments (TME); significant anti-metastatic effect	Feng et al. (2025)
Nucleic acid-based neoantigen	NP-mRNA + anti-CTLA-4	MUC1 mRNA	MRC1	4T1 subcutaneous tumor model of mice	Significant delay in tumor growth	Liu et al. (2018), Lin et al. (2022)
	PTC NVs@MNs	TdRNA	CpG ODN	4T1 tumor model of mice	Promotes dendritic cell maturation and activates T lymphocyte-mediated antitumor immunity; significant delay in tumor growth	Wang et al. (2025a)
Viral vector-based neoantigen	Neo-NV	Zfp142+ SpyTag003 (ST) peptide	——	4T1 cell xenograft tumor model of mice	Effectively suppressed tumor growth of orthotopic 4T1 breast tumor; significant prevention from tumor recurrence and metastasis	Zheng et al. (2023)

#### 3.2.1 Protein/peptide-based neoantigen cancer vaccines

Protein or peptide vaccines are currently the most widely used in neoantigen cancer vaccines (Dehghankhold et al., 2024). Compared with peptide vaccines, protein vaccines contain more antigenic epitope information, exhibit stronger immunogenicity and stability, and can elicit both CD8^+^ T and CD4^+^ T cell responses. During neoantigen sequencing alignment, differentially expressed proteins can be directly selected for vaccine design.

For example, autophagosomes obtained from the pleural effusions and ascites of cancer patients have been identified as a rich source of tumor neoantigens, exhibiting enhanced immunogenicity. Tumor cell-derived autophagosome (TRAPs) vaccines are enriched in autophagosome-packaged cellular proteins, defective ribosomal products (DRiPs), short-lived proteins (SLiPs), and surface markers. Compared to vaccines derived from whole tumor cells, they demonstrate greater efficacy in mediating tumor regression. Despite the immune enhancement induced by TRAPs, limitations remain, such as the suboptimal size for TRAPs endocytosis and their poor targeting to lymph nodes (LNs). Therefore, the effective delivery of antigens (Ag) and adjuvants to the LNs, where DCs maturation occurs, via targeted nanocarriers is required to enhance antigen cross-presentation and boost tumor-specific T cell responses. For instance, Qu et al. developed a bioinspired autophagosome-like nanovaccine (BAPs). This nanovaccine was formed by precisely integrating autophagosome-derived neoantigens and two lymph node-targeting adjuvants—the TLR-9 agonist CpG and the STING agonist ADU-S100—into self-assembled nanovesicles. By efficiently delivering antigens and adjuvants to lymph nodes, BAPs significantly enhances the maturation of antigen-presenting cells (DCs) and induces a broad T-cell response, particularly promoting the infiltration of CD8^+^ cytotoxic T lymphocytes (CTLs), which substantially inhibits tumor growth. In a 4T1 breast cancer mouse model, immunization with BAPs not only effectively eliminated tumors but also established long-lasting immune memory. When combined with immune checkpoint blockade (ICB) therapy, it markedly improved antitumor efficacy and extended mouse survival (Qu et al., 2024).

However, peptide vaccines feature well-defined sequences, ease of preparation and storage, and stable chemical properties. They can directly bind to MHC molecules without undergoing APC presentation, thereby activating T cells and eliciting robust CD8^+^ T cell responses. In contrast, conventional peptide antigens exhibit weak immunogenicity due to factors such as containing only a single epitope, small molecular weight, and susceptibility to degradation, which limits their anti-tumor efficacy. Therefore, adjuvants are typically employed or peptide vaccines are specifically designed to enhance their immunogenicity (Hirayama and Nishimura, 2016; Novakova et al., 2025).

For example, Zhou et al. constructed a nanovaccine termed Man-PDPM@OVA by loading the neoantigen synthetic peptide OVA (SIINFEKL) and the STING agonist 5,6-dimethylxanthenone-4-acetic acid (DMXAA) into micellar NPs. Since OVA (SIINFEKL) can be recognized by cytotoxic CD8^+^ T lymphocytes to elicit a specific immune response, Man-PDPM@OVA effectively accumulates in lymph nodes, enhances dendritic cell (DC) uptake, and promotes cytoplasmic release of the neoantigen, thereby significantly suppressing tumor growth and progression in both B16-OVA melanoma and 4T1 breast tumor models (Zhou et al., 2020). Similarly, Hu et al. conjugated the HER2-derived CH401 peptide on the outer surface of Physalis mottle virus (PhMV)-based VLPs and loaded a Toll-like receptor 9 (TLR9) agonist into the inner cavity of the VLPs, creating the nanovaccine CpG-PhMV-CH401. Upon administering CpG-PhMV-CH401 in a mouse tumor model, it is found that the nanovaccine significantly delay tumor growth and prolong survival in mice (Hu and Steinmetz, 2021). Feng et al. selected melittin (Mel), an active component of bee venom, as an inducer for *in situ* cancer vaccination, and ginsenoside Rh2 (Rh2) to remodel the tumor microenvironment (TME). By integrating tumor neoantigens (α-Mel-nAg) with melittin, they constructed a multicomponent biomimetic nanovaccine based on traditional Chinese medicine, termed nAg-MRDE/Mn. The nanovaccine achieves efficient uptake by tumor cells through the SR-BI receptor targeting by the D4F peptide, GLUT1 targeting by Rh2, and the membrane-penetrating effect of melittin (Mel). *In vitro* and *in vivo* studies demonstrated efficient cross-presentation of neoantigens, activation of immunogenic cell death (ICD) effects in tumor cells and NK cells via the cGAS-STING pathway, successful induction of neoantigen-specific immune responses, and improvement of the TME (Feng et al., 2025). These studies confirm that developing nanovaccines based on neoantigen peptides represents a promising platform for cancer vaccine development.

#### 3.2.2 Nucleic acid-based neoantigen cancer vaccines

Nucleic acid vaccines primarily include various forms such as DNA, RNA, and mRNA vaccines. Among these, neoantigen RNA vaccines represent a relatively novel class of cancer vaccines. They utilize mRNA to instruct cells to produce tumor-specific neoantigens, thereby triggering an immune response against the patient’s cancer cells. The neoantigen mRNA tumor vaccine works by delivering mRNA sequences carrying genetic information of tumor neoantigens to APCs. This enables APCs to use the engineered mRNA as a template to express and present relevant antigens, subsequently activating the immune system. Compared with traditional vaccines, mRNA tumor vaccines demonstrate several advantages: they are well-tolerated, readily degradable, and do not integrate into the host genome. Moreover, they possess the capability to induce both humoral and cell-mediated immune responses. Additionally, mRNA vaccines offer cost-effectiveness, rapid production timelines, and non-infectious properties, displaying promising prospects for clinical applications (Zhang et al., 2022; Li M. et al., 2024). For instance, Liu’s team delivered an NP-mRNA nanovaccine encoding the tumor neoantigen mucin 1 (MUC1) combined with mannose receptor C type 1 (MRC1) to stimulate the immune system. This was administered alongside anti-cytotoxic T-lymphocyte-associated antigen 4 (CTLA-4) immune checkpoint inhibitors to treat triple-negative breast cancer (TNBC). Results demonstrated that the NP-mRNA nanovaccine successfully expressed tumor antigens in DCs within lymph nodes, while simultaneously inducing a potent antigen-specific CTLs response against TNBC 4T1 cells *in vivo* (Liu et al., 2018). Subsequently, they also found that the combination therapy of NP-mRNA nanovaccine and anti-CTLA-4 monoclonal antibody significantly enhanced the therapeutic efficacy against TNBC by increasing CD8^+^ T cell infiltration into tumor sites, boosting anti-tumor cytotoxic T lymphocyte activity, reducing the immunosuppressive TME, and inhibiting the pro-tumorigenic STAT3 signaling pathway (Lin et al., 2022).

Furthermore, tumor-derived total RNA (TdRNA) vaccines demonstrate remarkable potential. They utilize total mRNA extracted from tumor cells to encode multiple tumor-specific antigens, activating T cells and B cells to elicit immune responses against these antigens. Additionally, exogenous RNA can be recognized by pattern recognition receptors (such as TLR3, TLR7, and TLR8) on DCs, promoting DC maturation and activation. This enhances the immune system’s ability to recognize and eliminate tumor cells. TdRNA vaccines induce broad immune responses by synthesizing tumor-specific antigens or activating pattern recognition receptors, making them a promising tool for activating CTLs in cancer immunotherapy. However, the inherent instability of RNA molecules causes them to degrade before reaching APCs, complicating the stimulation of specific anti-tumor immune responses. Although pulsing DCs with TdRNA is a feasible approach, it is costly and labor-intensive. To address this challenge, nanoparticle delivery systems—specifically, synthetic nanovaccines—have emerged as a promising strategy to enhance the stability of RNA tumor vaccines. For example, Wang et al. developed an efficient TdRNA nanovaccine loaded with negatively charged TdRNA and CpG oligodeoxynucleotides (CpG ODN), which was encapsulated in a hyaluronic acid (HA) microneedle matrix solution to create a dissolvable microneedle delivery platform termed PTC NVs@MNs. *In vitro* studies demonstrated that the TdRNA-loaded nanovaccine PTC NVs@MNs promoted the maturation of DCs, thereby activating an anti-tumor immune response. In mouse experiments, PTC NVs@MNs not only enhanced DC maturation but also increased the infiltration of CD8^+^ T cells into tumors, eliciting a robust anti-tumor immune response that effectively suppressed tumor growth while simultaneously inducing anti-tumor immune memory (Wang J. et al., 2025).

#### 3.2.3 Viral vector-based neoantigen cancer vaccines

Viral vector vaccines are prepared using replication-deficient or attenuated viruses as carriers. The viral proteins themselves can elicit an anti-tumor immune response. Due to their relatively mature development, favorable safety profile, and ability to induce a strong T-lymphocyte-specific immune response without the need for adjuvants, these vaccines are of significant importance in cancer immunotherapy (Nooraei et al., 2021).

Zheng et al. utilized SpyCatcher003 (SC)-modified norovirus S protein (Nov-S) NPs fused with the tumor neoantigen Zfp142 to prepare a virus-vector-based neoantigen nanovaccine (Neo-NV). The prepared neoantigen nanovaccine (Neo-NVs) could effectively target lymph nodes (LNs) and bind to DCs within LNs, triggering robust antigen-specific T cell immunity. This elicited potent systemic and local anti-tumor cellular immune responses in mice bearing orthotopic 4T1 breast tumors, significantly inhibiting tumor growth. Furthermore, Neo-NV immunization combined with PD-1 monoclonal antibody (mAb) therapy significantly prevented post-surgical tumor recurrence and metastasis. Notably, CTLs response levels in mice treated with anti-PD-1 mAb alone were similar to those in control mice. This implies that treatment with immune checkpoint inhibitors (ICIs) alone is insufficient to restore anti-tumor cellular immunity in immunologically “cold” tumors, while the combination therapy demonstrated a clear synergistic effect. Overall, this study provides a highly promising virus-vector-based neoantigen vaccine strategy and demonstrates the effectiveness of combined immune checkpoint blockade therapy in preventing post-surgical tumor recurrence and metastasis (Zheng et al., 2023).

### 3.3 Tumor cell membrane-biomimetic nanovaccines enhance targeted delivery

Another significant advantage of nanocarriers is their ability to be conjugated with targeting moieties. The surface of nanovaccines can be functionalized to enhance specific delivery to DCs. By modifying the nanovaccine surface with ligands for DC-specific surface receptors, targeted delivery of NPs to DCs can be achieved. These ligands include toll-like receptors (TLRs), C-type lectin receptors (CLRs) such as DEC-205, Clec9A, mannose receptor, and dendritic cell inhibitory receptor 2 (DCIR2), facilitating direct antigen presentation to DCs (Cruz et al., 2012).

**FIGURE 1:**
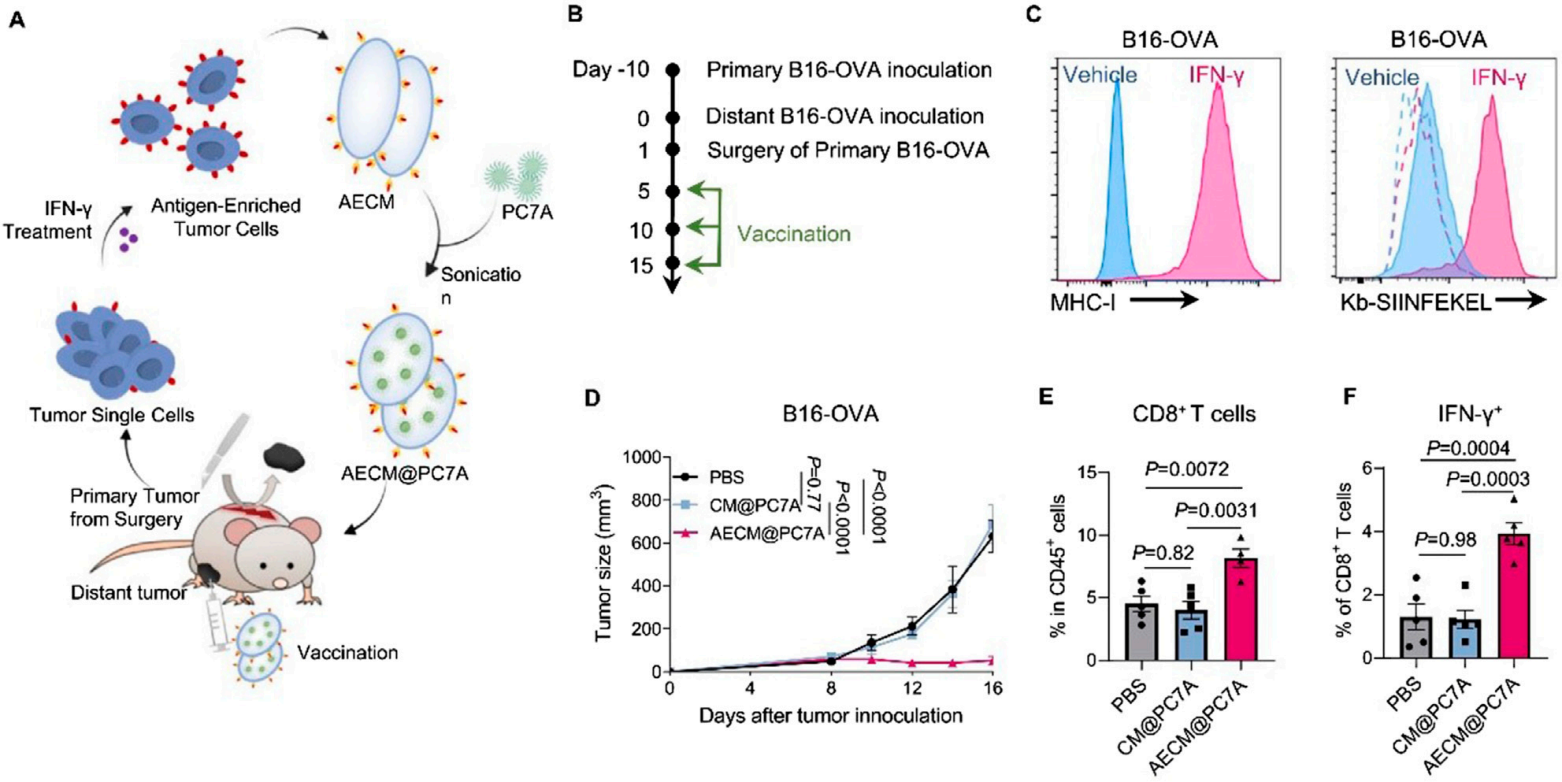
Anti-tumour effects of AECM@PC7A in post-surgical distant tumour model. Scheme **(A)** and timeline **(B)** to show the preparation of AECM@PC7A nanovaccine from surgically resected primary tumours for personalised treatment on distal tumours. **(C)** Representative histograms showing MHC-I expression on B16-OVA cells from resected primary tumours post IFN-γ treatment. Dashed lines, isotype; solid lines, anti-Kb-SIINFEKEL. **(D)** Distant tumour growth curves by different vaccinations. **(E)** Tumour-infiltrating CD8^+^ T-cells at day-16 post distant tumour inoculation. **(F)** The IFN-γ secretion by CD8^+^ T-cells of PBMCs after re-stimulation with OVA257-264 peptide was determined by flow cytometry. Adapted with permission from (Li et al., 2025), copyright 2025, Springer Nature.

Although nanomedicine-based drug delivery systems offer many advantages, the exogenous nature of nanoparticles makes them susceptible to recognition and elimination by the immune system. PEGylation technology has been widely used to reduce the clearance rate of nanoparticles. However, reports indicate that PEGylated nanoparticles can induce anti-PEG antibodies upon repeated administration, which paradoxically makes these nanoparticles more easily cleared by the immune system [ ]. Lipids, as major components of cell membranes, have also been applied to prepare biomimetic liposomes that mimic biological membranes. However, their structural lack of integrity and stability limits their application as nanovaccine delivery systems [ ]. With the rapid development of biomimetic drug systems and antigen delivery technology, tumor cell membrane biomimetics with micro-nano dimensions have garnered significant attention. As a mature biomimetic drug modification technique, tumor cell membrane coating has been widely used to modify nanomaterials, endowing non-biological materials with good biocompatibility, specific tumor targeting, and immune-activating properties (Li et al., 2023; Wang W. et al., 2024; Yang et al., 2024). The cell membrane is rich in tumor-associated antigens and devoid of genetic material, making it easy to modify or reassemble. Therefore, tumor cell membrane-coated drug delivery systems demonstrate unique advantages, enabling the co-delivery of tumor antigens with various functional molecules such as chemotherapeutic drugs, photosensitizers, radiosensitizers, and immunomodulators, thereby exerting combined immunotherapeutic anti-tumor effects. Particularly, tumor cells that have undergone immunogenic cell death (ICD) exhibit greatly enhanced immunogenicity, along with significantly increased exposure of calreticulin (CRT) on their cell surface. Nanovaccines based on ICD tumor cell membrane coating can induce efficient active uptake by DCs, enhancing anti-tumor efficacy. Tumor cell membrane-based biomimetic nanocarriers have demonstrated significant promise in the design of breast cancer vaccines (Table 3). For instance, Xiao et al. utilized 4T1 cancer cell membranes (CCM) to coat poly (lactic-co-glycolic acid) (PLGA) NPs loaded with R837 (an agonist of Toll-like receptor 7), constructing a biomimetic cancer cell membrane nanovaccine (designated as CCMP@R837). The CCMP@R837 nanovaccine enhanced the uptake and maturation of bone marrow-derived DCs and increased the antitumor response against 4T1 breast cancer cells *in vitro*. After immunizing BALB/c mice three times with CCMP@R837, immune memory was established. When the immunized mice were challenged with 4T1 cancer cells, CCMP@R837 recognized and destroyed the cancer cells by increasing CD8^+^ T cells and reducing regulatory T cells in the tumor, thereby achieving durable antitumor immunity. Consequently, this biomimetic nanovaccine constructed using tumor cell membranes demonstrates significant potential for the development of nanovaccines for cancer immunotherapy (Xiao et al., 2021). Li et al. constructed a novel multifunctional bionic nanovaccine, MP@RHM. This vaccine was fabricated by enveloping manganese oxide-loaded poly (2-diisopropylaminoethyl methacrylate) (MP) nanoparticles with hybrid cell membranes derived from MnO_2_-reprogrammed 4T1 cells and DCs, designed for combined chemodynamic-immunotherapy. Compared to single-cell-membrane-coated nanovaccines, MP@RHM efficiently activates DCs and T cells through the synergistic effects of abundant damage-associated molecular patterns (DAMPs), Mn^2+^, and T-cell-stimulating components. Following peritumoral injection, MP@RHM not only targets tumor sites for chemodynamic therapy but also homes to lymph nodes to potently activate tumor-specific T cells, thereby achieving chemodynamic-immunotherapy (Li T. et al., 2024). Li et al. pretreated tumor cells with IFN-γ to develop a biomimetic nanovaccine called AECM@PC7A, based on antigen-enriched tumor cell membranes (AECM). AECM@PC7A can directly “transfer” the entire MHC-I antigen complex from the tumor cell membrane to the surface of DCs, thereby activating T cells. As a result, the AECM@PC7A vaccine can induce a robust tumor-specific CD8^+^ T cell response even at low doses. In mouse models, the AECM@PC7A nanovaccine significantly inhibited tumor growth and metastasis, extended survival, and elicited long-term immune memory protection. Furthermore, using a humanized immune system CDX (patient-derived xenograft) model, it was demonstrated that the AECM@PC7A nanovaccine also activates a strong human CD8^+^ T cell response, leading to tumor regression in 40% of mice with MDA-MB-231 human breast cancer (Figure 1) (Li et al., 2025).

**TABLE 3:** Modification strategies for enhancing efficient delivery of nanovaccines.

Modification strategies	Nanovaccines	Vaccine antigen	Adjuvant	Tumor model	Outcome	References
Tumor cell membrane-biomimetic nanovaccines	CCMP@R837	4T1 cancer cell membrane (CCM)	R837	4T1 subcutaneous tumor model of mice	Significant suppression tumor progression; prolonged survival period	Xiao et al. (2021)
	MP@RHM	hybrid cell membrane (RHM) derived from manganese oxide-remodeled 4T1 cells and dendritic cells (DCs)	——	mouse tumor model	Effectively elicit durable immune memory T cells to inhibit postoperative tumor metastasis and recurrence	Li et al. (2024b)
	AECM@PC7A	antigen-enriched cell membrane	PC7A	MDA-MB-231 cell xenograft tumor model of mice	Completely protect mice from tumor challenge; significantly suppress the tumor growth	Li et al. (2025)
	CM-CpG-aCD40+anti-PD-L1	4T1 CCM	CpG ODN + aCD40	4T1 subcutaneous tumor model of mice	Effectively suppressed the tumor progression and prolonged the survival time in TNBC-bearing mice	Yan et al. (2025)
pH-sensitive nanovaccines	CaCO_3_@TCL/CpG	tumor cell lysates (TCL)	CpG ODN	4T1 subcutaneous tumor model of mice	pH enhance infiltration of immune cells and activate antitumor immune response; significantly inhibits tumor growth of tumor-bearing mice	Ding et al. (2023)
	dPEDE-A@M32+anti-PD-1	——	ADU-S100	4T1 subcutaneous tumor model of mice	Effectively control drug release and co-delivery in an acidic environment; effectively inhibited tumor growth; suppressed recurrent and metastatic tumor progression	Wang et al. (2025b)
Targeting cancer stem cells	(ALDH-A1-TMR)-ND + anti-PD-L1	ALDH1-A1 epitopes	CpG ODN	4T1 subcutaneous tumor model of mice	Generated robust ALDH-specific T cell responses; significant suppression tumor growth; prolonged survival period	Hassani Najafabadi et al. (2020)
	NICER	ALDH1A1	siYthdf1	4T1 subcutaneous tumor model of mice	Significantly inhibit tumor proliferation and tumor recurrence and metastasis mediated by tumor stem cells	You et al. (2025)

Furthermore, various cell membrane vesicles derived from organisms, particularly those from macrophages and tumor cells, are highly suitable as carriers for antitumor drugs due to their dual capabilities of immune evasion and active targeting. For instance, Yan et al. developed a B-cell-targeting tumor nanovaccine, CM-CpG-aCD40, by conjugating two immune enhancers—an agonistic CD40 antibody (aCD40) and CpG—onto tumor cell membrane vesicles using a small-molecule crosslinking agent. CM-CpG-aCD40 can actively target and accumulate in peripheral immune organs. Through the binding of antigens on the tumor cell membrane to B-cell receptors, the interaction of aCD40 with CD40, and the binding of CpG to Toll-like receptor 9 (TLR9), it provides multiple activation signals to B cells, enhancing their antibody secretion and antigen-presenting capabilities. This nanovaccine also promotes dendritic cell maturation to activate CD8^+^ T cells and reprograms tumor-associated macrophages toward an M1 phenotype. By comprehensively activating humoral, cellular, and innate immunity, the vaccine achieved an 89.3% tumor growth inhibition rate in a TNBC mouse model (Yan et al., 2025).

### 3.4 pH-sensitive nanovaccines facilitate tumor-microenvironment-targeted delivery

**FIGURE 2:**
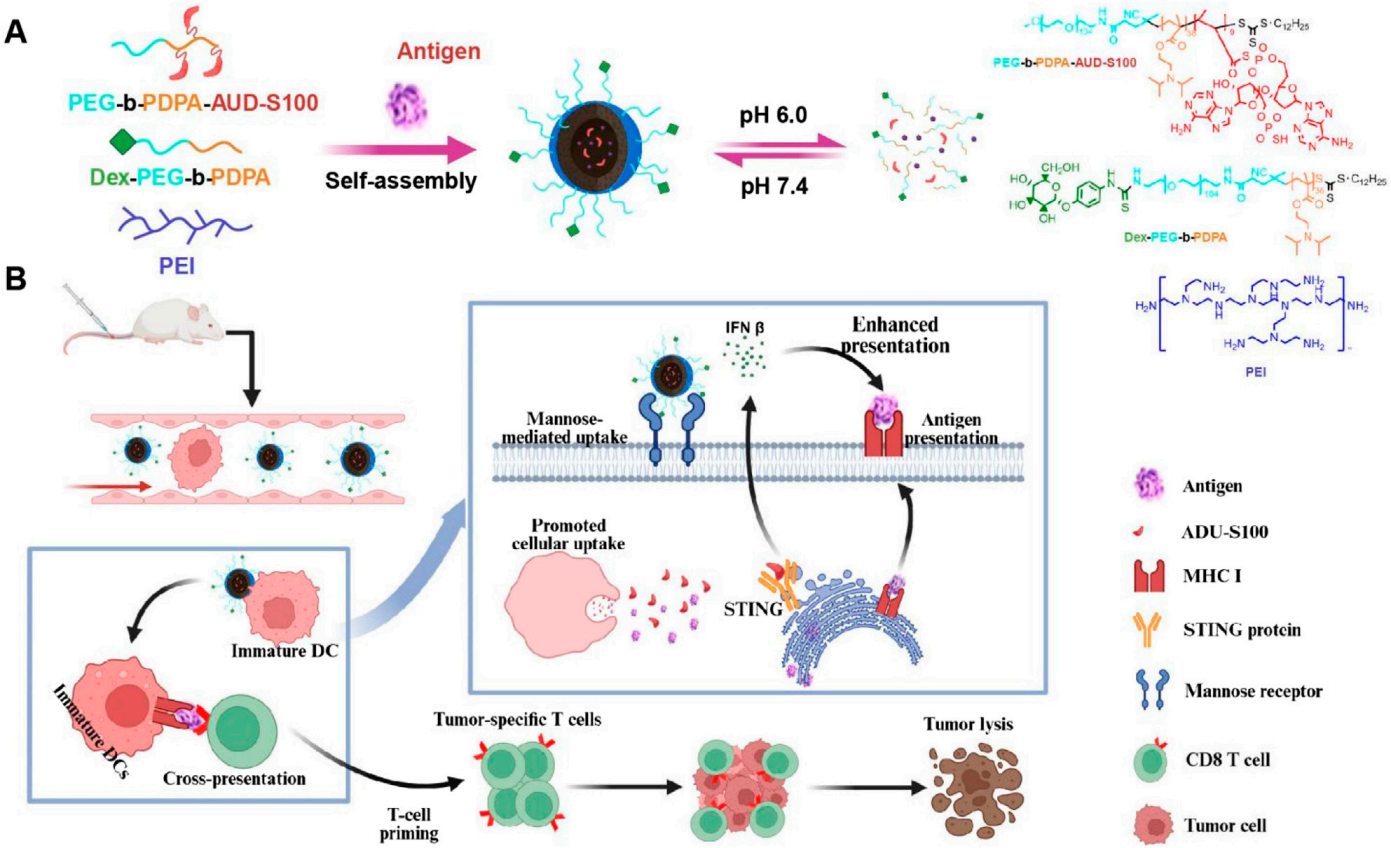
Schematic of the nanovaccine delivering STING agonist and antigen. **(A)** Schematic illustration of the nanovaccine preparation and disassembly process of the nanovaccine; **(B)** Diagram illustrating how the nanovaccine enhances the STING pathway and boosts T-cell immune responses to improve immunity. Adapted with permission from (Wang N. et al., 2025), copyright 2025, Ivyspring International Publisher.

To enhance the delivery efficiency of nanomaterials, nanocarrier systems can also be engineered as intelligent carriers with tumor microenvironment responsiveness or stimulus responsiveness, enabling controlled drug release. This advantage can significantly reduce premature drug release from the nanocarrier before reaching the tumor site, thereby decreasing drug accumulation in non-tumor tissues and consequently lowering drug-related systemic toxicity. These nanovaccines responsive to endogenous stimuli within the tumor microenvironment not only facilitate the delivery of nanodrugs to tumors but also control drug release at the tumor site. This enables passive tumor targeting, enhances therapeutic efficacy, and reduces systemic toxicity. For instance, the pH of the breast cancer microenvironment (6.5–7.0) is lower than that of normal tissues and blood. Exploiting this characteristic acidic environment of breast cancer tissues or cells, a series of acid-sensitive nanodrug delivery systems can be constructed for breast cancer treatment (Harris et al., 2024; Kundu et al., 2024). Nanoscale Drug Delivery Systems (NDDS) containing ester bonds, carboxyl groups, acetals, imidazole groups, amide bonds, and boronate esters have been demonstrated to possess pH-responsive drug release properties. These pH-sensitive chemical bonds remain structurally stable under physiological conditions but undergo hydrolytic cleavage in acidic environments (Table 3). This accelerates drug release from the nanoscale prodrugs, achieving high local drug concentrations at tumor sites and thereby helping to maximize therapeutic efficacy. Ding et al. constructed a pH-sensitive nanovaccine based on calcium carbonate (CaCO_3_) nanoparticles. This nanocarrier was co-loaded with tumor cell lysate (TCL) and the immune adjuvant CpG (CpG oligodeoxynucleotide 1826), forming the pH-responsive nanovaccine CaCO_3_@TCL/CpG for TNBC immunotherapy. The CaCO_3_@TCL/CpG nanovaccine decomposes in the acidic tumor microenvironment (TME) or within endolysosomes after endocytosis. This decomposition enables the simultaneous delivery of tumor-associated antigens (TAAs) and the immune adjuvant to DCs, significantly enhancing antigen presentation to T cells and triggering a potent anti-tumor response. Moreover, CaCO_3_ may help reverse the immunosuppressive TME by consuming excess hydrogen ions and lactate. This reversal increases the M1/M2 macrophage ratio, thereby enhancing the immune response against TNBC and recruiting cytotoxic T cells to further kill tumor cells. Furthermore, the nanovaccine significantly inhibited tumor growth in a subcutaneous 4T1 tumor model in tumor-bearing mice (Ding et al., 2023). Wang et al. developed a novel pH-responsive targeted nanovaccine based on nanotechnology, dPEDE-A@M32. Due to the presence of tertiary amine (-N (iPr)_2_) groups in the 2-(diisopropylamino)ethyl methacrylate (DPA) component of this nanovaccine, protonation occurs under acidic conditions, enabling pH-responsive drug release. Additionally, dPEDE-A@M32 encapsulates a STING agonist and a neoantigen, allowing it to specifically activate DCs and trigger a robust T-cell immune response. In mouse models, dPEDE-A@M32 effectively inhibited tumor growth, induced antigen-specific T-cell responses, and suppressed tumor recurrence and metastatic progression. Combination therapy with an anti-PD-1 antibody further enhanced tumor control, immune cell infiltration, and survival rates. This nanotechnology-based pH-responsive nanovaccine not only improves treatment specificity but also enhances immune responses by modulating the TME (Figure 2) (Wang N. et al., 2025). Overall, this pH-sensitive nanovaccine represents a promising nanoplatform for enhancing BC immunotherapy.

### 3.5 Targeting cancer stem cells in nanovaccines design

**FIGURE 3:**
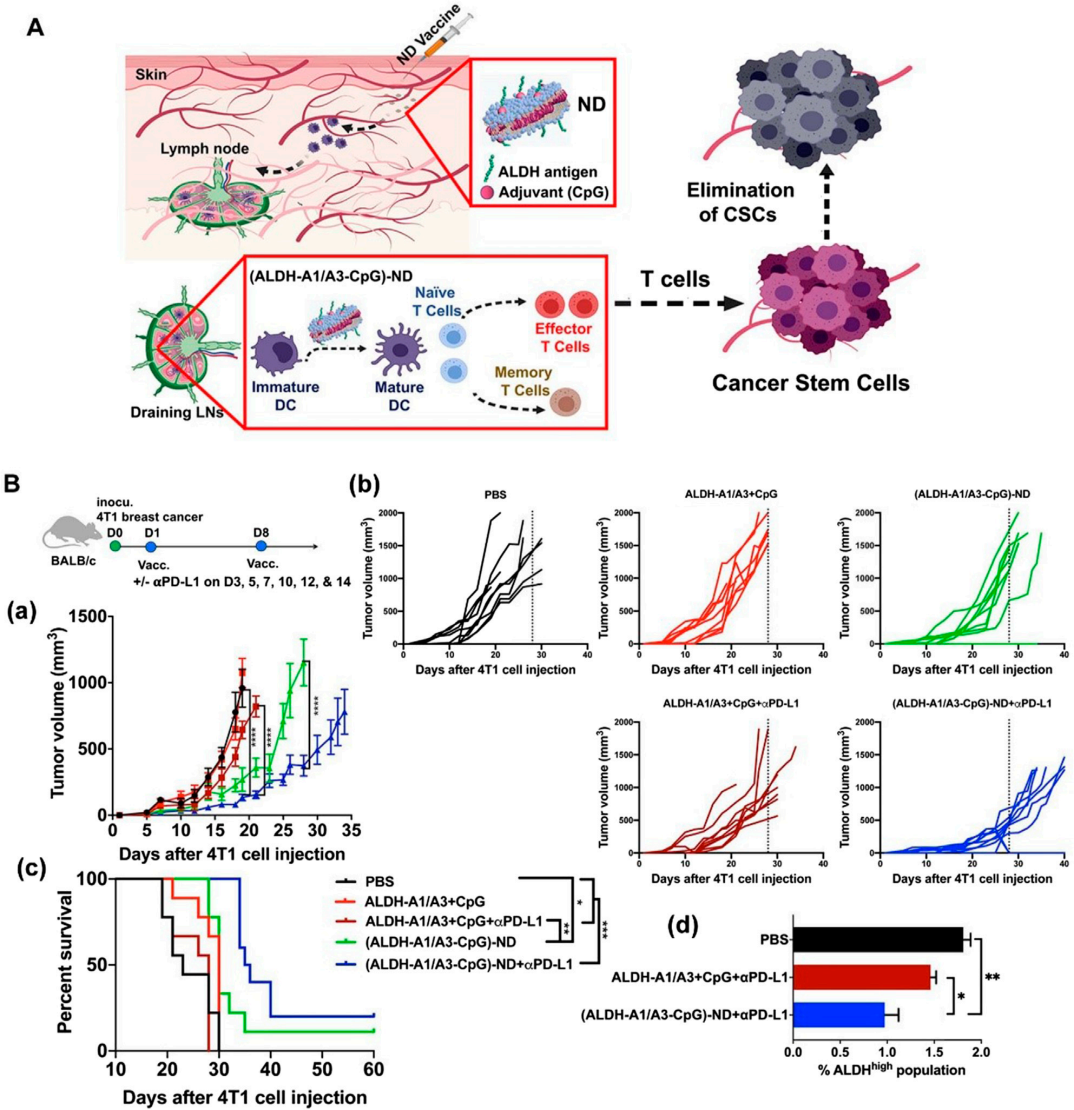
Vaccination against cancer stem cells (CSCs) with nanodiscs (NDs) carrying ALDH epitopes. **(A)** Schematic of ND vaccination against ALDHhigh CSCs. **(B)** Therapeutic efficacy of ALDH-ND vaccination in a 4T1 breast cancer model. (a) The average tumor growth, (b) individual tumor growth, and (c) overall animal survival. (d) Frequencies of ALDHhigh CSCs among live cells within residual 4T1 tumor masses. Adapted with permission from (Hassani Najafabadi et al., 2020), copyright 2020, American Chemical Society.

Another major challenge in BC treatment is postoperative recurrence and metastasis. Even after radical surgical resection, residual microscopic lesions or circulating tumor cells can still lead to disease recurrence. Particularly, the presence of cancer stem cells (CSCs), considered the “seeds” of recurrence and metastasis, poses a significant threat. These cells possess self-renewal and differentiation capabilities, enabling them to enter a dormant state to evade elimination under therapeutic pressure, only to resume proliferation and form new tumors when conditions become favorable (Gupta et al., 2022). While conventional chemotherapy and targeted therapies can kill the majority of tumor cells, their effectiveness against CSCs is limited, making it difficult to eradicate the risk of recurrence. Even if 95%–99% of tumor cells are eliminated, the residual 1%–5% of cancer stem cells can still cause the disease to relapse. Therefore, tracking and eradicating residual CSCs after surgery is crucial for preventing tumor recurrence. To address the challenge of breast cancer recurrence, nanovaccines targeting CSCs have emerged as a prominent research focus (Table 3) (Hua et al., 2022; Kumar et al., 2023). Najafabadi et al. identified the A1 and A3 epitopes of aldehyde dehydrogenase (ALDH) from CSCs and developed synthetic high-density lipoprotein (HDL) nano-discs for vaccination targeting ALDH1A3-high (ALDHhigh) CSCs. When combined with anti-PD-L1 IgG therapy, the nano-disc vaccination reduced the frequency of ALDHhigh CSCs in tumor tissue and exerted potent anti-tumor effects against multiple tumors known to contain CSCs. In mouse models of melanoma and breast cancer, the ALDHhigh CSC-targeting nano-disc vaccine combined with anti-PD-L1 therapy demonstrated robust anti-tumor efficacy and prolonged animal survival. Overall, this represents an innovation in simple nanovaccine strategies targeting CSCs and may offer a novel approach for CSC-targeting cancer immunotherapy (Figure 3) (Hassani Najafabadi et al., 2020). You et al. developed an epigenetically regulated antigen-integrated nanovaccine (NICER) based on tumor cell-derived nanovesicles co-expressing tumor-associated antigens and tumor stem cell-specific antigens. This vaccine enables simultaneous immune clearance of both tumor cells and CSCs. Specifically, nanovesicles derived from tumors overexpressing aldehyde dehydrogenase (ALDH1A1) serve as integrated antigens carrying CSC-specific antigens and tumor-associated antigens, thereby inducing immune responses against both ALDHhigh CSCs and differentiated tumor cells. The study demonstrated that by targeting YTHDF1 (YTH N6-methyladenosine RNA binding protein 1), a methyl reader protein in DCs, the vaccine effectively reduces lysosomal protease expression in DCs. This reduction decreases the degradation of integrated antigens and enhances antigen cross-presentation efficiency. Compared to the no-vaccine treatment group, the vaccine achieved a 1- to 2-fold increase in antigen cross-presentation efficiency, significantly boosting its immunogenic potency. Concurrently, the vaccine markedly inhibited tumor proliferation and suppressed CSC-mediated tumor recurrence and metastasis. Compared to the no-vaccine group, the vaccine increased the tumor inhibition rate by approximately 5- to 7-fold (You et al., 2025).

## 4 Combination therapy strategies

Although tumor vaccines can initiate APC-mediated antigen-specific T cell immune responses, during the effector phase, the tumor immunosuppressive microenvironment hinders the activation of effector lymphocytes and diminishes their ability to infiltrate the TME. Furthermore, immunosuppressive cells such as tumor-associated macrophages (TAMs), myeloid-derived suppressor cells (MDSCs), and cancer-associated fibroblasts (CAFs) counteract the function of immune effector cells by expressing immunosuppressive receptors or secreting immunosuppressive cytokines. Given that single-agent tumor vaccines struggle to overcome the immunosuppressive microenvironment, leading to low clinical response rates, researchers have proposed various combination therapy strategies. These strategies integrate tumor vaccines with other therapies like photothermal therapy (PTT) to achieve robust activation and functional modulation of the immune system, thereby controlling tumor progression. The combination of nanovaccines with novel therapeutic modalities such as photothermal therapy can generate synergistic effects, ultimately enhancing the efficacy of tumor treatment (Table 4).

**TABLE 4:** Combination therapy strategies.

Therapy strategies	Nanovaccines		Vaccine antigen	Adjuvant	Tumor model	Outcome	References
Nanovaccine-photothermal therapy	iDP-NS + PD-L1	Photosensitizer indocyanine green (ICG)	TAAs	CpG ODN	4T1 postoperative model of mice	Effectively trigger long immune memory to inhibit tumor recurrence and metastasis	Yu et al. (2022)
	BCNCCM	Photosensitizer BP-Au	indoleamine 2,3-dioxygenase (IDO) inhibitor (NLG919)	CpG ODN	Orthotopic 4T1-tumor model of mice	Effectively suppresses primary tumors growth and metastatic tumors	Huang et al. (2022)
	NIR-II NV	Photosensitizer TBS	The whole 4T1 tumor cells antigen	——	Orthotopic 4T1-tumor model of mice	Effectively enhance the antitumor immune response *in vivo*; significantly inhibited the proliferation and metastasis of breast cancer cells in the lung	Gao et al. (2025)
	PDA/ICG/CpG	Photosensitizer PDA/ICG	——	CpG ODN	Orthotopic xenograft 4T1 cells tumor model of mice	The cell viability of 4T1 and MDA-MB-231 cells decreased significantly; outstanding tumor suppression; significantly restrain tumor growth and inhibit splenic metastasis *in vivo*	Hu et al. (2025b)
Nanovaccine-sonodynamic therapy	G5-CHC-R	Sonosensitizer Chenghai chlorin (CHC)	——	resiquimod (R848)	Orthotopic 4T1-tumor model of mice	Notably induced tumor regression	Wang et al. (2024b)
Nanovaccine-nanozyme therapy	Au/CuNDs-R848	Catalase (CAT)-like activity	——	R848	Orthotopic 4T1-tumor model of mice	Significantly inhibited primary tumor growth; significantly restrict the growth of distant tumors	Wang et al. (2024d)
	Cu SAZ	Peroxidase (POD)-like activity	——	——	4T1 subcutaneous tumor model of mice	Significantly enhanced survival; significant increase in tumor-infiltrating CD8^+^ T cells; significantly inhibited tumor growth	Ye et al. (2025)
	GSNO@B	Glucose oxidase (GOx)- like activity	——	——	Orthotopic 4T1-tumor model of mice	Effectively suppresses primary tumors growth and metastatic tumors	Hu et al. (2025a)
	HABH	GOx- and POD-like activities	——	——	Orthotopic 4T1-tumor model of mice	Eradicate primary tumors and prevent metastasis tumors	Xu et al. (2025)

### 4.1 Synergistic nanovaccine-photothermal therapy strategies

**FIGURE 4:**
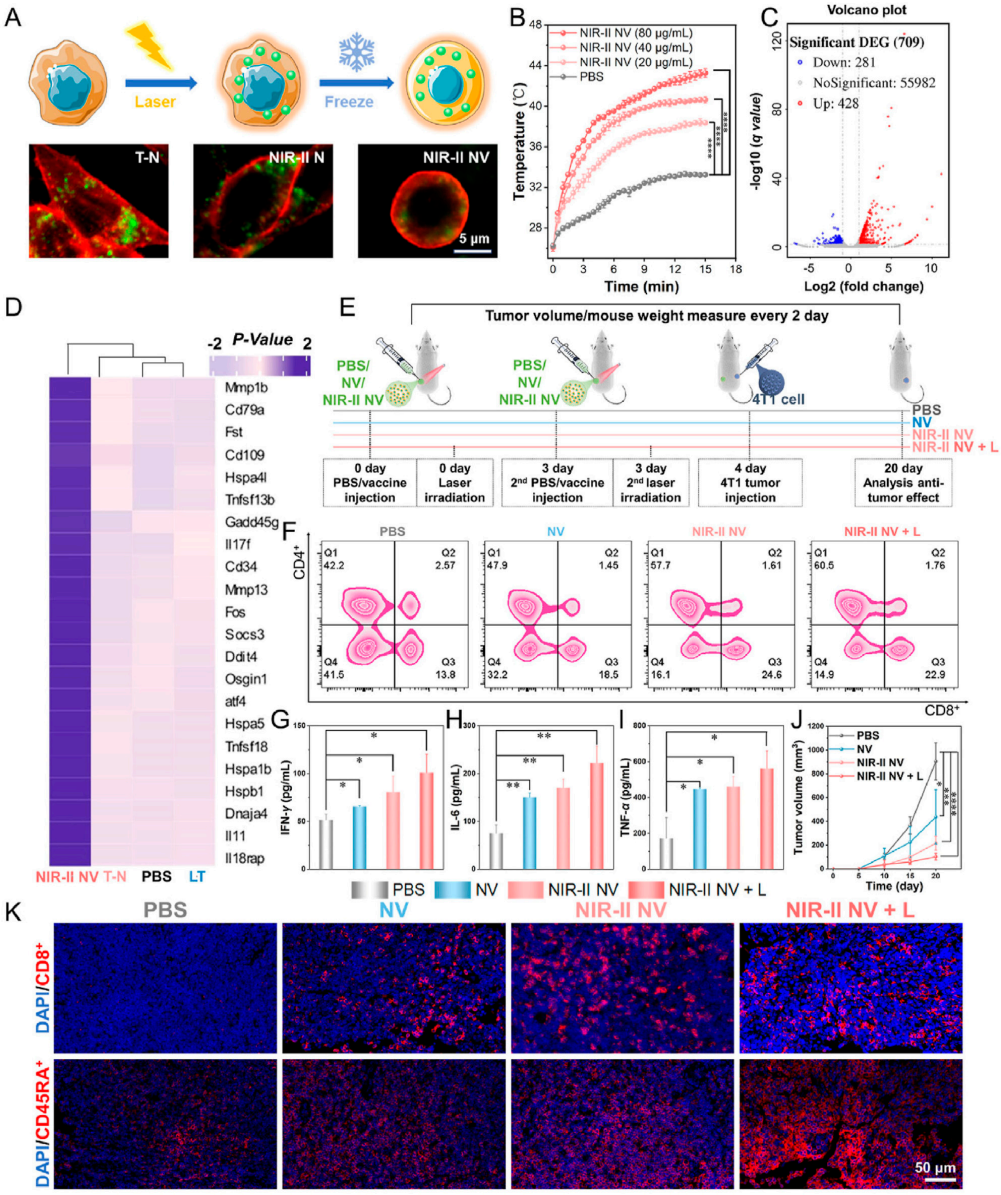
*In vivo* tumor treatment of NIR-II activated vaccine. **(A)** Vaccine preparation and cell imaging of the NIR-II NV construct. **(B)** Temperature changes of the NIR-II NVs (10^7^ cells/mL, 500 μL) after incubation with different concentrations of TBS NPs exposed to 15 min laser irradiation of 1,064 nm (1 W/cm2). **(C)** Volcano plot of all DEGs in the NIR-II NV-treated cells. **(D)** Heatmap of differential gene cluster analysis of the treated cells. **(E)** Schematic diagram of tumor prevention by the NIR-II NV-based vaccine. **(F)** Analysis of CD4^+^ T and CD8^+^ T cells in the lymph nodes of mice on the 4th day after treatment. **(G–I)** Analysis of **(G)** IFN-γ, **(H)** IL-6, and **(I)** TNF-α in mouse serum on the 4th day after treatment. **(J)** Tumor volume changes of the treated mice within the treatment. **(K)** Infiltration levels of CD8^+^ T cells (cytotoxic T cells) and CD45RA + T cells (memory T cells) in the tumor sites on the 20th day posttreatment. Adapted with permission from (Gao et al., 2025), copyright 2025, Elsevier BV.

PTT has been recognized as one of the cancer treatment methods with promising development prospects in recent years. Compared to traditional therapies, PTT has garnered increasing attention due to its advantages of high selectivity, non-invasiveness, minimal drug resistance development, oxygen independence, and reduced side effects. PTT refers to a treatment method that utilizes materials with excellent photothermal conversion efficiency to transform light energy into heat energy, thereby raising the temperature of surrounding tissues to induce apoptosis or necrosis in cancer cells. Studies have shown that tumor cells have a lower tolerance to high temperatures compared to normal cells. Under laser irradiation, tumor cells undergo cell lysis and enzyme release, leading to necrosis and protein denaturation, thereby achieving effective tumor cell killing. Simultaneously, PTT can further enhance its anti-tumor efficacy by increasing blood flow in tumor tissue, improving oxygenation within the tumor microenvironment, and enhancing the infiltration of immune cells into the tumor (Kadkhoda et al., 2022). However, single-modality cancer therapies currently have certain limitations. For instance, while PTT can inhibit primary tumors through thermal ablation, it is often unable to completely eradicate tumors or effectively prevent metastasis and recurrence. This is due to factors such as limited light penetration depth, reduced photothermal efficiency caused by photobleaching after short-term laser irradiation, and inefficient treatment resulting from significant oxygen dependency. Conversely, the efficacy of immunotherapy is often diminished by the weak immunogenicity of tumors and the immunosuppressive tumor microenvironment. Therefore, the combination of photothermal therapy and immunotherapy has attracted growing interest, with the expectation that it can address their respective limitations and achieve satisfactory synergistic therapeutic outcomes (Table 4) (Kong and Chen, 2022). Yu et al. synthesized DNA-photosensitizer nanospheres (iDP-NS) using the immune adjuvant CpG ODN and the 808 nm photosensitizer ICG (which triggers both photodynamic and photothermal reactions upon laser irradiation). These nanospheres were used in combination with PD-L1 blockade to exert anti-tumor effects. The hybrid nanosphere-mediated dual-modal photothermal/photodynamic therapy (PTT/PDT) served to release TAAs and deliver the immune agonist CpG-ODN to DCs, promoting their maturation and migration to lymph nodes. This enhanced antigen presentation. Concurrently, synergistic immune checkpoint blockade therapy reactivated cytotoxic T cell activity, thereby generating a potent immune response. This combined approach could eliminate primary tumors, trigger robust immune memory, and effectively suppress postoperative metastasis and recurrence in the 4T1 model (Yu et al., 2022). Huang et al. co-encapsulated black phosphorus-gold (BP-Au) nanosheets loaded with the immune adjuvant CpG oligodeoxynucleotide (ODN) and the indoleamine 2,3-dioxygenase (IDO) inhibitor (NLG919) within a cancer cell membrane (CCM). This process yielded a CCM-based *in situ* photothermal nanovaccine (BCNCCM) designed for the elimination of primary and metastatic breast tumors. The nanovaccine leverages the targeting capability of CCM to selectively accumulate at the tumor site. Following PTT, it exhibits a vaccine-like function through the combined action of *in situ*-generated tumor-associated factors and the immune adjuvant CpG, thereby triggering tumor-specific immunity. Furthermore, the IDO inhibitor mediates the reversal of the immunosuppressive microenvironment, further enhancing the tumor-suppressive effect. Experimental results demonstrated that this nanovaccine not only exhibits potent therapeutic efficacy against both primary and metastatic tumors but also prevents tumor recurrence by generating systemic immune memory (Huang et al., 2022). Gao et al. designed a nanovaccine based on semiconducting bi-base oligomer TBS NPs, modified with polyethyleneimine (PEI), constructing the nanovaccine NIR-II NV. This vaccine possesses NIR-II activation capability, enabling it to activate anti-tumor immune memory and effectively inhibit tumorigenesis and metastasis *in vivo*. Research results showed that under 1,064 nm light excitation, NIR-II NV effectively induced tumor cell apoptosis and the release of immunogenic proteins. Furthermore, the role of nanovaccine NIR-II NV in preventing tumorigenesis was investigated in a 4T1 tumor xenograft mouse model. The results indicated that NIR-II NV pre-activated with 1,064 nm light effectively prevented tumors, and 1,064 nm light irradiation of the tumor site further enhanced the preventive efficacy of NIR-II NV. Simultaneously, they also investigated the potential of NIR-II NV to prevent tumor metastasis. After two vaccinations and local 1,064 nm light irradiation, a lung metastasis mouse model was established by intravenous injection of 4T1 cells. The results demonstrated that NIR-II NV significantly suppressed the proliferation and metastasis of breast cancer cells in the lungs. These results confirm the effective preventive and inhibitory effects of the NIR-II-based nanovaccine NIR-II NV on tumor metastasis (Figure 4) (Gao et al., 2025). Hu et al. utilized polydopamine (PDA) as a photosensitizer and loaded the imaging agent indocyanine green (ICG) and the adjuvant CpG ODN onto the surface of PDA nanospheres, respectively, to prepare the nanovaccine PDA/ICG/CpG (PIC). They innovatively employed an intervaginal space injection (ISI) administration strategy, enabling targeted delivery to tumor tissues and draining lymph nodes while bypassing the bloodstream. Following ISI injection, PDA/ICG/CpG precisely accumulated at the tumor site. Under laser irradiation, tumor cells were eradicated via PTT, releasing tumor-associated antigens that promoted DCs maturation and CD8^+^ T-cell infiltration. The PTT-enhanced immune response mediated by combination immunotherapy facilitated the accumulation of mature DCs and CTLs at the tumor site, as well as their trafficking to lymph nodes and the spleen. This effectively suppressed tumor growth in the 4T1 breast cancer model and inhibited splenic metastasis (Hu N. et al., 2025). Thus, these studies demonstrate that combining PTT with nanovaccine immunotherapy represents a promising strategy for BC treatment.

### 4.2 Synergistic nanovaccine-sonodynamic therapy strategies

**FIGURE 5:**
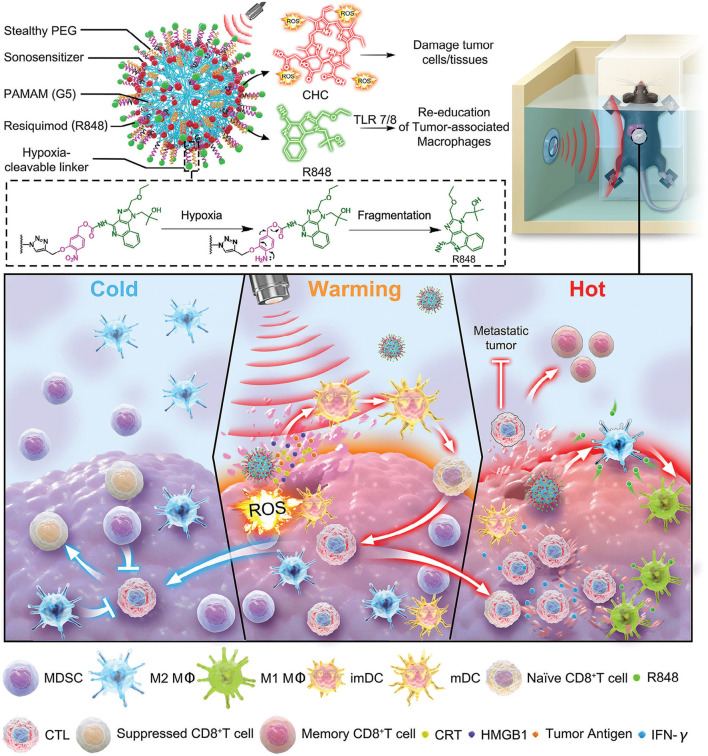
Schematic illustration of the structure of the sono-nanovaccines and their therapeutical mechanisms driven by the free-field based whole-body US irradiation. Scheme of the sono-nanovaccines, G5-CHC-R (upper), and schematic diagram of the sono-nanovaccines for direct tumor cells killing as well as amplifying the cascade of the immune responses via “cold-warm-hot” three-state transformation of TME (lower). Adapted with permission from (Wang et al., 2024b), copyright 2024, Wiley-VCH Verlag.

Although therapeutic nanovaccines for tumors have achieved remarkable success, cancer immunotherapy offers limited clinical benefits to patients with solid tumors due to their immunosuppressive microenvironment. Poorly immunogenic solid tumors, characterized by deficient host-intrinsic antitumor immunity and insufficient T-lymphocyte infiltration—termed immunologically “cold” tumors—are prone to evade immune clearance and drive progression and metastasis by altering their local microenvironment. Therefore, generating vaccines *in situ* at the tumor site using the full spectrum of tumor antigens is considered a promising strategy. Following certain treatments such as chemotherapy or radiotherapy, the induction of immunogenic cell death (ICD) within the tumor region can achieve an ideal *in situ* vaccination effect by promoting the release of TAAs and triggering antigen-specific cytotoxic T-cell immune responses. Among all ICD-inducing therapeutic modalities, sonodynamic therapy (SDT) stands out as a superior option. It employs low-intensity ultrasound (US) to activate sonosensitizers, generating reactive oxygen species (ROS) that induce tumor cell apoptosis or necrosis, thereby facilitating localized exposure of diverse tumor-associated antigens. The combination of nanovaccines with SDT represents a cutting-edge approach in current tumor immunotherapy. By synergistically activating immune responses and physically modulating the tumor microenvironment, it significantly enhances antitumor efficacy (Son et al., 2020; Yang et al., 2023). The synergy between nanovaccines and SDT offers a breakthrough direction for solid tumor treatment through a tripartite strategy of “physical destruction–immune activation–microenvironment remodeling” (Table 4). For example, Wang et al. constructed an SDT-synergized nanovaccine platform called G5-CHC-R by conjugating the sonosensitizer porphyrin derivative Chenghai chlorin (CHC)—which exhibits high sonosensitizing activity—and the immunomodulator resiquimod (R848) onto a generation 5 polyamidoamine (PAMAM, G5) dendritic macromolecular nanoscaffold. In this G5-CHC-R nanovaccine, the immunomodulator R848 is conjugated to G5 via a hypoxia-sensitive linker and can thus be cleaved by nitroreductase (NTR), which is highly expressed in tumor tissues, enabling the localized release of R848 at the tumor site. Under systemic free-field US irradiation, the sonodynamic nanovaccine G5-CHC-R generates reactive oxygen species (ROS), inducing ICD and TAAs release, thereby converting immunologically “cold” tumors into immune-responsive “hot” tumors. Upon entering the tumor, under hypoxic conditions, R848 can be released from its hypoxia-sensitive linker, further enhancing antitumor immunity by reprogramming tumor-associated macrophages (TAMs), suppressing myeloid-derived suppressor cells (MDSCs), and boosting cytotoxic CD8^+^ T-cell responses. This ultimately transforms the tumor into a “hot” phenotype. In two murine models—one with orthotopic pancreatic cancer accompanied by intestinal metastasis and another with breast cancer with lung metastasis—the sonodynamic nanovaccine significantly controlled primary tumor growth and distant metastasis by achieving a “cold-to-hot-to-hot” triphasic transformation within the tumor microenvironment (TME), thereby potentiating systemic antitumor immune responses. Additionally, G5-CHC-R demonstrated the ability to prevent tumor recurrence by inducing durable immune memory (Figure 5) (Wang et al., 2024b).

### 4.3 Synergistic nanovaccine-nanozyme therapy strategies

Nanozymes are a class of materials that possess both the unique properties of nanomaterials and catalytic functions mimicking natural enzymes. In tumor therapy, nanozymes primarily function as oxidoreductases. Studies indicate that tumor development leads to redox dysregulation in the tumor microenvironment. By mimicking natural enzymes such as superoxide dismutase (SOD)-like, peroxidase-like, and catalase-like activities, nanozymes can modulate oxidative stress levels, promote the generation of reactive oxygen species (ROS), and induce tumor cell death, thereby inhibiting tumor progression. When synergized with immunotherapy, nanozymes significantly enhance immunotherapeutic efficacy through multiple mechanisms: augmenting antigen presentation; activating immune cells; and remodeling the tumor microenvironment. They empower the immune system to better recognize and attack cancer cells while enhancing the potency of immune cells (Table 4) (Phan et al., 2022; Wang et al., 2023).

**FIGURE 6:**
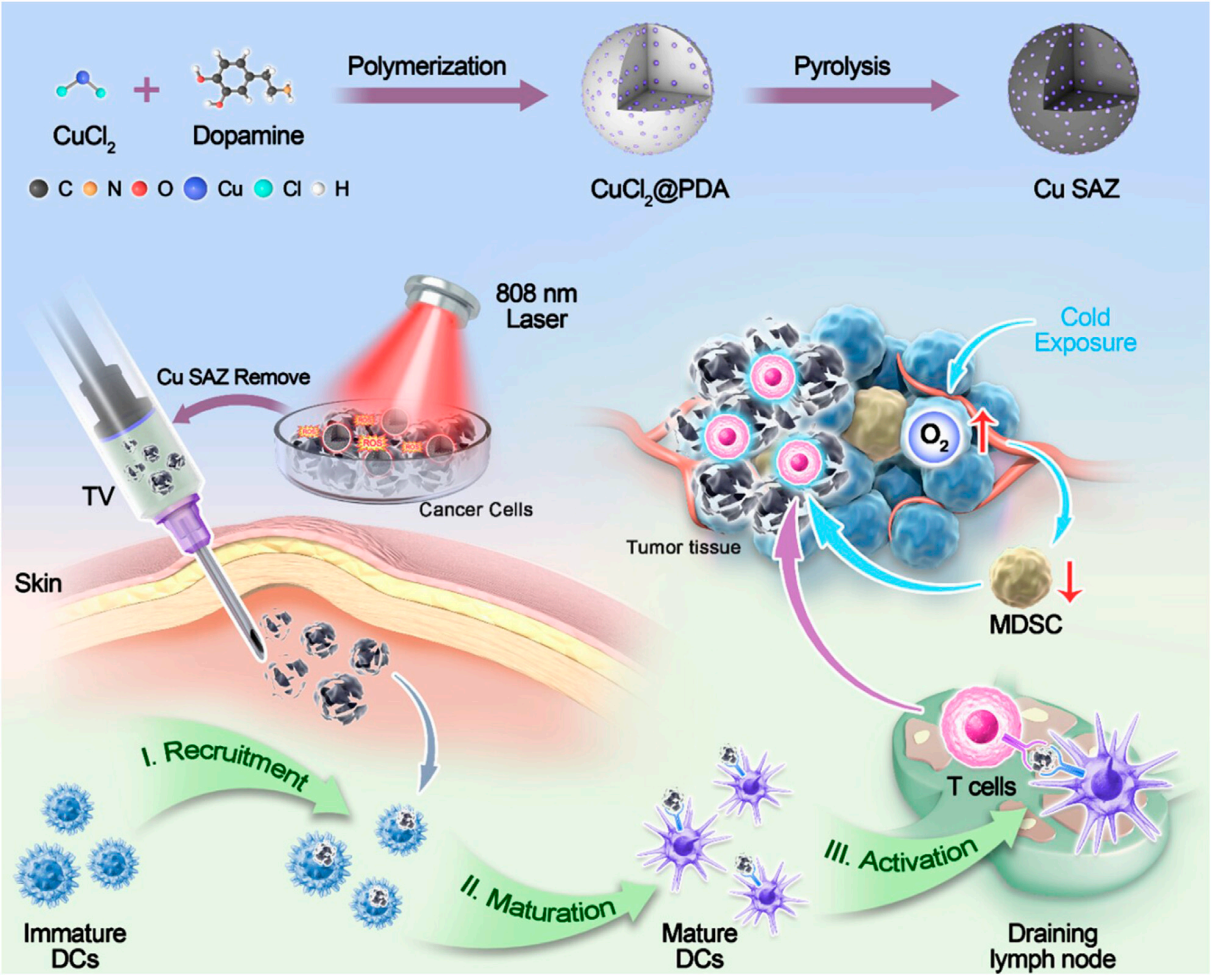
Schematic illustration of cold exposure therapy enhancing single-atom nanozyme-mediated tumor vaccines therapy. Adapted with permission from (Ye et al., 2025), copyright 2025, American Chemical Society.

Wang et al. designed an “*in situ* nanovaccine” named Au/CuNDs-R848. This nanovaccine features Au/CuNDs with excellent photothermal properties and nanozyme activity, and incorporates the immune adjuvant R848 to enhance immune responses. Studies demonstrate that Au/CuNDs-R848 catalyzes the decomposition of H_2_O_2_ to generate hydroxyl radicals (·OH). Additionally, Au/CuNDs-R848 significantly depletes intracellular glutathione (GSH) in tumor cells, further enhancing the efficacy of chemodynamic therapy (CDT). Through the synergistic action of PTT and CDT, the Au/CuNDs-R848 nanovaccine not only promotes the maturation of DCs and enhances the infiltration of T lymphocytes but also successfully induces immunogenic cell death (ICD) in 4T1 cells. Through dual immunomodulation, “cold tumors” were transformed into “hot tumors,” significantly inhibiting *in vivo* tumor growth and enhancing immunotherapy for metastatic TNBC (Wang Z. et al., 2024). Ye et al. synthesized a copper single-atom nanozyme (Cu SAZ) via high-temperature carbonization. This material exhibits excellent peroxidase (POD)-like activity and photothermal properties, with its long-term antitumor efficacy further enhanced by incorporating cold exposure (CE) therapy. Cold exposure therapy—a non-invasive and direct antitumor approach—not only suppresses tumor metabolism but also ameliorates the immunosuppressive tumor microenvironment. Studies demonstrate that under 808 nm laser irradiation, the nanozyme generates ROS and heat, inducing immunogenic cell death in 4T1 breast cancer cells or CT26 colon cancer cells. *In vivo* studies reveal that CE significantly potentiates the therapeutic effect by reducing the proportion of myeloid-derived suppressor cells (MDSCs), suppressing tumor cell glycolysis, and increasing the proportions of CD8^+^ T cells and memory T cells. This combined approach effectively inhibits tumor growth (Figure 6) (Ye et al., 2025). Hu et al. designed and developed a tumor microenvironment (TME)-responsive self-assembled nanozyme named GSNO@B. This nanozyme is formed through the self-assembly of a nitric oxide (NO) donor-modified glucose oxidase (GOx) with the hydrophobic immune checkpoint inhibitor BMS-202. Within the TME, GSNO@B specifically dissociates to release the enzymatically active GSNO. On one hand, GOx consumes glucose, reducing platelet production, suppressing their activity, and diminishing microaggregate formation. On the other hand, the released NO further weakens the protective adhesion of platelets to tumor cell surfaces and disrupts microaggregates, thereby exposing tumor cells to blood shear forces and immune cell recognition. This dual action effectively prevents the formation of platelet-tumor cell complexes, inhibiting both the physical migration and immune evasion of tumor cells. Concurrently, the immune checkpoint inhibitor BMS-202, released from the dissociated GSNO@B, blocks the formation of PD-1/PD-L1 complexes. This enhances the ability of effector T cells to recognize and eliminate circulating tumor cells (CTCs), further promoting the immune clearance of CTCs. Both *in vitro* and *in vivo* experiments demonstrate that the GSNO@B nanozyme effectively suppresses primary tumor growth and significantly reduces metastatic tumor formation. This indicates its potential to robustly inhibit breast cancer progression by blocking platelet-mediated hematogenous metastasis pathways. This study provides a safe and promising adjuvant therapeutic strategy for metastatic tumors, offering new hope for breast cancer patients and opening novel avenues for cancer treatment (Hu B. et al., 2025). Xu et al. developed a nanozyme-active tumor vaccine named HABH by loading a BODIPY-derived type I photosensitizer (BDP) into gold nanoparticle-engineered hollow mesoporous silica nanoparticles (HMSN/AuNPs), followed by surface coating with hyaluronic acid (HA). HABH targets CD44-overexpressing tumor cells via HA. Upon cellular internalization, it releases BDP. Under light irradiation, BDP performs type I photodynamic therapy (PDT), generating abundant OH. This induces mitochondrial stress and damage, leading to the release of mitochondrial DNA (mtDNA) into the cytoplasm and subsequent activation of the cGAS-STING pathway. Simultaneously, HABH exhibits dual nanozyme activities (glucose oxidase-like and peroxidase-like). After entering cells, it catalyzes the oxidation of glucose to hydrogen peroxide (H_2_O_2_) and further oxidizes the generated H_2_O_2_ into highly toxic hydroxyl radicals (·OH). Under hypoxic conditions, this intensifies intracellular oxidative stress. Combined with the mitochondrial stress induced by BDP, it ultimately triggers the cGAS-STING pathway, promoting the release of type I interferons. In an orthotopic breast tumor model, HABH not only inhibited primary tumor growth but also effectively prevented tumor metastasis. In conclusion, this study provides novel insights into nanozyme-synergized photoimmunotherapy for BC (Xu et al., 2025).

## 5 Conclusions and prospects

Nanovaccines, as an emerging strategy for breast cancer immunotherapy, offer a novel therapeutic avenue to address the challenges in treating TNBC and combating postoperative recurrence. This is achieved through precise delivery of tumor antigens, efficient activation of immune responses, and remodeling of the tumor microenvironment. The use of nanovaccines can significantly enhance the immunogenicity of molecular vaccines. They effectively co-deliver multivalent molecular antigens and adjuvants to lymphoid tissues and immune cells, thereby boosting antigen-specific adaptive immune responses during cancer treatment. By leveraging various nanoengineering technologies, diverse nanovaccines have been developed and applied in cancer immunotherapy. Tumor nanovaccines can effectively treat cancer by appropriately delivering tumor antigens to APCs. This leads to the maturation and activation of APCs, increases the infiltration of anti-tumor functional CD8^+^ T cells, promotes DCs activation and the formation of antigen repertoires, and maintains stability. These effects collectively contribute to maximizing the potential of cancer therapeutic vaccines. In this review, we systematically summarize various nanomaterial carriers used in the development of nanovaccines, such as liposome NPs, inorganic NPs, polymer NPs, and biomimetic membrane NPs. These nanomaterials exhibit excellent biocompatibility, adjuvant activity, and immunogenicity. Furthermore, we discuss and summarize preparation strategies to enhance antitumor immune efficacy. These include the application of tumor neoantigens to improve antigen presentation, the use of tumor cell membrane biomimetic nanotechnology to promote targeted delivery of nanovaccines, nanovaccine delivery platforms targeting the TME, and novel strategies for combination therapies with nanovaccines. These innovative approaches to nanovaccine preparation are expected to have a profound impact on the treatment of cancers such as BC (Davodabadi et al., 2022; Desai et al., 2024; Wang et al., 2024c).

However, the unique characteristics of the TME present both opportunities and challenges for the treatment of BC. Due to the rapid growth of tumors and the abnormalization of blood vessels, insufficient blood supply often occurs within the tumor, leading to long-term hypoxia. Increased cellular metabolism results in the accumulation of lactate and hydrogen ions, thereby creating an acidic TME. The TME can provide targets for drug delivery design to enhance the therapeutic effects, particularly through TME-responsive nanodelivery systems, which demonstrate great potential in targeted antitumor delivery and controlled drug release. Nevertheless, the existence of the TME also poses significant challenges for antitumor therapy using nanovaccines. For instance, advanced BC microenvironments harbor multiple immunosuppressive mechanisms. These include substantial infiltration of immunosuppressive cells (such as Tregs, MDSCs, and TAMs), which secrete inhibitory factors (e.g., TGF-β, IL-10) that suppress T-cell function. Consequently, the resulting immunosuppressive state of the tumor microenvironment may limit vaccine efficacy. Furthermore, the dense stromal matrix in breast cancer may impede intratumoral penetration and cellular uptake of nanovaccines. Therefore, developing stroma-remodeling nanocarriers or utilizing physical methods such as focused ultrasound to enhance delivery constitutes a potential solution. Currently, although intelligent TME-responsive nanodelivery systems are in a rapid stage of development, several challenges remain, and their design strategies still require improvement. For instance, the biocompatibility and drug-loading capacity of intelligent nanovaccines need further enhancement. Poor biocompatibility or low drug-loading capacity may lead to suboptimal therapeutic efficacy and even induce tumor drug resistance. The use of naturally derived cell membrane-coated biomimetic nanomaterials or ligand modifications could improve their biocompatibility, targeting specificity, and drug-loading capacity. Additionally, nanovaccines designed solely based on a single characteristic of the TME—such as intrinsic stimuli like high GSH and pH-related enzymes—often exhibit limited therapeutic efficacy and inherent constraints. Combining these with external stimuli, such as temperature, sound, light, magnetic fields, and ions, could create synergistic effects and more effectively inhibit tumor growth. Furthermore, developing matrix-remodeling nanocarriers or utilizing physical methods like focused ultrasound to enhance delivery represents another potential solution. Additionally, developing matrix-remodeling nanocarriers or utilizing physical methods such as focused ultrasound to enhance delivery also represents a potential solution (Harris et al., 2024; Kundu et al., 2024).

Future research in breast cancer nanovaccines will advance in the following directions: (a) Exploring novel antigen strategies: Investigating approaches such as shared neoantigens, fusion antigens, optimized antigen epitopes, and mRNA-encoded multi-antigens to enhance immunogenicity and coverage. Furthermore, combining tumor mRNA vaccines with immune checkpoint inhibitors (such as anti-PD-L1 antibodies) or other immunostimulants represents a combinatorial strategy that helps overcome immunosuppression in the tumor microenvironment. This approach leverages nanovaccines to amplify neoantigen presentation, enhance T cell immune responses, and synergize with immune checkpoint blockade, thereby further improving antitumor efficacy (Jin et al., 2024; Zheng et al., 2024). (b) Integrating new materials and technologies: Leveraging AI-assisted antigen design, biodegradable smart materials for sustained antigen release, and multifunctional nanocarriers to achieve integrated diagnostics and therapy. Designing “personalized” nanomaterials and implementing “personalized” treatments based on tumor heterogeneity and TME heterogeneity is expected to enhance the effectiveness and applicability of smart nanomaterials in the TME. (c) Optimizing vaccine delivery systems: Developing novel adjuvant systems (e.g., manganese nano-adjuvants), refining lymph node-targeting strategies (e.g., surface charge modulation), and designing multi-stage delivery systems (e.g., stimuli-responsive release materials). (d) Development of novel biomimetic nanovaccines: Biomimetic nanovaccines offer benefits such as targeted delivery to APCs, improved loading of antigens/adjuvants, and enhanced biocompatibility, thereby increasing sensitivity to immunotherapy (Xu et al., 2024). Particularly, artificial APCs (aAPCs) are designed to mimic the cellular functions of natural APCs, triggering the activation and expansion of antigen-specific T cells (Yang et al., 2020). By incorporating APC-targeting molecules into biomimetic nanovaccines, the delivery efficiency of these vaccines can be effectively enhanced (Liu et al., 2021). (e) Expanding clinical research: Promoting the entry of more nanovaccines into clinical trials for breast cancer, particularly in early-stage neoadjuvant therapy and postoperative adjuvant therapy.

Future studies must focus on addressing translational bottlenecks, including individualized vaccine manufacturing timelines, standardized production, and clinical application protocols. Concurrently, in-depth exploration of synergistic mechanisms between nanovaccines and immune checkpoint inhibitors, radiotherapy/chemotherapy, and other emerging therapies is essential. With the deep integration of nanotechnology, immunology, and genetic engineering, breast cancer nanovaccines are poised to transition from the laboratory to the clinic. This progress holds promise for ultimately benefiting a broad patient population—especially those with TNBC lacking effective treatment options—bringing the vision of precision immunotherapy to fruition.
